# Hannover study on long-stay hospitalization – part I: prediction of long-stay hospitalisation in cases of chronic mental illness

**DOI:** 10.1186/1745-0179-2-10

**Published:** 2006-05-22

**Authors:** Stefan M Bartusch, Hermann Elgeti, Peter Bastiaan, Wielant Machleidt, Marc Ziegenbein

**Affiliations:** 1 Department of Social Psychiatry and Psychotherapy, Hannover Medical School, 30623 Hannover, Germany

## Abstract

**Background:**

The problem of long-stay hospitalization is still a pressing issue. In this study we examined the possibility of detecting and characterising the group at risk of long-stay hospitalization in advance.

**Methods:**

This study examines the data of patients in the urban catchment area of the Medical University of Hannover, capital of Lower Saxony, Germany, during a period of 10 years.

**Results and conclusion:**

The introduced "psychosocial risk-score", calculated at the first institutional contact, was able to predict the risk of long-term hospitalization. Characteristics of social disintegration, especially with regard to employment status, are of particular importance.

## Introduction

A survey of the literature of the past 100 years referring to schizophrenia [1] makes apparent the impossibility to predict the individual course of the illness. We know from many long-term studies that positive courses occur more frequently than assumed. According to Wing [2], intensive and repeated studies have as yet been unsuccessful in tracing predictors for primary prevention. However, considerable knowledge is available about useful techniques for secondary and tertiary prevention. Important implements of such secondary prevention are: Considering the severity of the disorder, the frequency of relapses and the development of chronic deficits, its identification and treatment; securing best social conditions; encouraging the patient to gain a sufficient understanding of the disease, to reduce risk factors like stress and to accept help from local care services; providing sufficient care services.

Studies concerning the course and the prognosis of the disease are of specific significance in the research of schizophrenia. Comparing four German long-term studies, Marneros [3] shows that the great range of results as to the "outcome" of schizophrenic psychoses can – among other reasons – be explained by a non-standardised use of the term schizophrenia. The figures referring to the criteria complete remission or healing differ between 7 and 30 percent. Comparison of the "Cologne-study" [4] and the "Bonn-study" [5] shows complete remissions in approximately 1/4 to 1/5 of the examined group, development into "untypical residual states" in nearly half of the group and development of "typical residual states" in 1/3 to 1/4 of the group [3]. In the course of long-term studies, too, the period of observation has also to be considered. Whereas it is certainly possible after five years to state whether or not chronic defects have developed, this period is too short to be definite about the kind and seriousness of alterations of the disease, or about one's autarchy and the necessity of permanent hospitalization [3].

Many studies have been conducted to try to find predictors for the course of schizophrenic disorders, in order to distinguish disadvantageous from advantageous courses [6]. However, the studies proved discrepant and sometimes even showed contradictory results. As Marneros [3] argued, the discrepancy could be explained by the fact that the outcome of the disease is not determined by a fixed state at a given time but rather by a continuum. Therefore, the outcome is multi-dimensional. He also stressed that the modification of the outcome by long-term treatment has not yet been examined sufficiently. In a review of the literature, Hubschmid and Ciompi [7] mentioned the significance of the following predictors as sufficiently verified: A generally good premorbid adjustment, a harmonious premorbid personality and an acute beginning of the disease indicate a positive course of the disease. In contrast, a lingering onset, distinct negativism and a poor response to medical treatment usually predict a rather negative course. Hereditary factors, neuropathological findings and florid symptoms, on the other hand, are of only limited significance.

There is sufficient literary evidence of a connection between unemployment and psychiatric disorders. For example, unemployment figures correlate directly with the rate of psychiatric hospitalizations in New York within a period of 127 years [8] as well as with the total utilisation of psychiatric care institutions in Northern Italy [9] and Great Britain [10]. Unemployment causes psychotic relapses [11,12], leads to chronicity [13] and is closely related to the number of so-called new long-stay psychiatric patients [14]. Mastroeni et al. showed, that the treatment provided prior to transfer from long-stay hospital to community residence may have long-term clinical benefits for chronically disabled patients [15].

As primary prevention of schizophrenia is presently not within reach, psychiatric care services in the community are of particular importance. It would therefore be good to detect from early on patients who are expected to have a negative course of the disease in the long run, rather than an early remission; these persons could be supplied with effective care offers at an early stage [16]. Our approach was to develop a working model, whose adoption into instruments of communal psychiatric planning may be possible. Such a procedure would be useful for detecting unfulfilled needs in the care system as well as for providing a preventive orientation towards the launching of communal psychiatric support actions.

## Material and methods

### The area and institutions

The examined area of this study is the psychiatric catchment area (Sector 5) of Hannover (capital of Lower-Saxony, Germany) served by the Department of Social Psychiatry and Psychotherapy of the Medical University of Hannover since the early seventies [17]. Hannover has approximately 500,000 inhabitants living in an area of 204 square kilometres. Sector 5 is the smallest area of all 10 sectors of the regional community psychiatric network, but is densely populated. From 1977 to 1997 the population decreased from 75,000 to 63,000 inhabitants. A characterisation of the social structure based upon the data of a census in 1987 [18] shows higher-than-average urban density and an above-average social situation.

The study group included patients and clients of the following institutions: the responsible psychiatric policlinic as an outpatient institution, the psychiatric department of a general hospital (Medical University of Hannover) and a complementary society which runs two residential homes and provides a sheltered workshop and occupational rehabilitation for mentally ill people. In 1991 the share of patients with a residence in the evaluated area was 87% in the policlinic, 31% in the psychiatric hospital and 11% in the complementary society.

Since the beginning of psychiatric reformatory efforts, these institutions have played an important role in the development of outpatient and complementary services in Hannover. The Social Psychiatric Policlinic of the Medical University of Hannover integrates the tasks of an official community psychiatric service and of a medical treatment centre. Psychotherapeutic, sociotherapeutic, pharmacological and rehabilitational treatment methods are applied in close co-operation with general practitioners, specialists, social institutions and the authorities.

### The study group

The study group was constituted by the following criteria:

- contact to one of the institutions included in the study (see above) in the period of 1987 until 1996,

- a period of at least two years between the first and the last time of contact in one of the three institutions mentioned (independent of a continuous use of the services),

- age between 18 and 60 years (at first contact),

- residence within the catchment area (Sector 5 of Hannover) at the time of first contact,

- first psychiatric diagnosis excluding addictions or psycho-organic disorders (e.g. dementia or mental handicap).

The archives of medical records of the Social Psychiatric Policlinic provide the most important source of data, as it was the institution which most of the patients (92%) had contacted. Additionally, the complete patients' records of the other two institutions were also evaluated. In the case of disputable data, the responsible therapists were consulted or the medical records from corresponding in-patient treatments were viewed.

For each patient of the study group the following anonymous characteristics were collected in a database:

Invariable data: sex, date of birth, year of onset of psychiatric disease, year of first contact to one of the institutions mentioned, basic data of the time of first contact (in the case of treatment in a residential home or hospital: before first contact), in cases where the treatment ended during the period of the study: year and reason of termination of treatment.

Variable data: basic data of each year of contact (first diagnosis, duration since onset of disease, living situation, work situation, primary income, address of residence), data of service of each year of contact: method and duration of institutional psychiatric supports (number of quarters for outpatient and complementary supports, number of days for the time of partial and full-time in-patient treatment).

The variable data were collected for each year between 1987 and 1996 in which at least one contact with the evaluated institutions had occurred. If the treatment was continued into 1997, the complete data of 1997 were registered. In cases with the first contact before 1987, the basic data of the year of first contact was also included in the database.

### The psychosocial risk score (PSR)

Using the characteristic features of the psychiatric base data, we composed a scaled sum score (psychosocial risk score = PSR; scale 1 to 4) for each patient and each year. We were interested in the predictive power of such a score for a subsequent long-stay hospitalization. The choice of the characteristic features was determined by the following considerations: first, the most precise indicators for the severity and chronicity of the psychiatric disorder and the dimensions of social exclusion relating to the risk of a future long-stay hospitalization were to be located in accordance with the empirical and scientific knowledge mentioned above. Furthermore, the characteristics were chosen according to their feasibility of data collection in everyday treatment. If the predictive power of the PSR for a subsequent long-stay hospitalization can be proven, an adoption of the PSR into instruments of communal psychiatric planning may be possible. Such a procedure would be useful for detecting unfulfilled needs in the care system as well as for providing a preventive orientation towards the launching of communal psychiatric support actions.

The following base data were chosen as possible indicators of the chronicity and severity of psychiatric disorder: the first psychiatric diagnosis, the age of onset of disease, the duration since onset of disease.

The degree of social disintegration was characterised by the following criteria: living situation, work situation, primary income.

The intensity of these six characteristics is recorded on a scale from 1 to 4, resulting in a sum of 6 to 24. These sum figures were divided into four groups with different psychosocial risks:

- sum score 6 to 10: low risk ( = 1),

- sum score 11 to 15: moderate risk ( = 2),

- sum score 16 to 20: significant risk ( = 3),

- sum score 21 to 24: high risk ( = 4).

For lack of a definition of long-stay hospitalization in the literature, we considered a person to be long-stay hospitalised if he or she matched at least one of the following criteria during the period of the study:

- duration of more than 365 days of in-patient treatment within two successive years (independent of the number of in-patient stays),

- living in a psychiatric residential home for at least four quarters within two successive years,

- transfer to an in-patient nursing institution.

All the data recorded were entered into SPSS for windows (version 12.0) and evaluated using the available statistical procedures such as descriptive analysis, non-parametric tests (Kruskal-Wallis test, Wilcoxon signed rank test, Mann-Whitney) U-test and correlation calculations (correlation analysis by Spearman). Significant differences were found when p < 0.05, and high significance was assumed when p < 0.01.

## Results

### Total group

Data of 313 patients were included in the study. This population is mainly female (62%) and had an average age of 48 years in the middle of the examination period. The majority (83%) lived within the catchment area at the time of first contact. 68% of the study group had the diagnosis of a non-organic psychotic disorder. The average age at the time of onset of disease was 30 years. Within a period of 6 years after onset, the patients were admitted to one of the evaluated therapeutic institutions. At this time, the majority (56%) were self-supporting, living together with at least one person in a flat, 46% were unemployed, 47% had a full-time job, one third of the population were receiving their own income, 26% were claiming insurance payments like unemployment benefits or pension, 27% were supported by relatives, 13% were on social security. At the time of the first contact, the average PSR was 2.3.

Nearly half of the examined patients (46%) were treated in one of the institutions even after the study period. In 21% the treatment was finished or terminated early, 17% were referred to other therapists, 6% of the group moved into another catchment area. In 10% a disadvantageous outcome of treatment was found: 21 patients had to move into a residential nursing home; 8 patients died within the period of the study, including 5 patients by suicide. The PSR *at the time of first contact *shows a low value (2.0) for the patient group who were living in their own flat at the end of the study. Above-average values were found in subgroups treated in external in-patient institutions (2.7) and in patients who died during the period of the study (Suicide: 3.0, other causes of death: 2.7).

### Group with long-stay hospitalization (table 1)

**Table 1:** Group with long-stay hospitalization (LSH) vs. total group

	group with LSH	total group
	
	22% (N = 68)	100% (N = 313)
	
Sex ratio *		
- female	47%	62%
- male	53%	38%
Residence at first therapeutical contact **		
- in catchment area	68%	83%
- outside catchment area	32%	17%

First diagnosis at first therapeutical contact		
- ICD-10: F4	6%	16%
- ICD-10: F6	12%	16%
- ICD-10: F2, F30.1, F30.2, F31, F32.2, F32.3, F33.2, F33.3	82%	68%

Period between onset of disease and first therapeutical contact^n.s.^		
- average	5 years	6 years
- < 1 year	25%	31%
- 1 until < 5 years	32%	26%
- 5 until < 10 years	13%	17%
- >10 years	30%	26%

PSR at first therapeutical contact **	2.6	2.3
- average	6%	16%
- low psychosocial risk	31%	43%
- moderate psychosocial risk	57%	38%
- significant psychosocial risk	6%	3%
- high psychosocial risk		

age of onset of disease **		
- average	27 years	30 years
- < 25 years	56%	38%
- 25 – 44 years	7%	51%
- 45 – 59 years	37%	11%

Employment situation at first therapeutical contact **		
- full-time job	28%	47%
- part-time employment	0%	4%
- in therapeutical institution	7%	4%
- unemployed	65%	46%

Primary income at first contact **		
- own income	10%	34%
- insurance payments	30%	26%
- relatives	35%	27%
- on social security	25%	13%

Living situation at first therapeutical contact^n.s. ^	52%	56%
- self-supporting, not alone	52%	56%
- self-supporting, alone	29%	37%
- in therapeutical institution	16%	5%
- homeless	3%	2%

* p < 0.05 ** p < 0.01 ^n.s. ^not significant (p > 0.05)

The group with LSH includes 22% (N = 68) of the study group. The ratio of women is only 47 % (in the total group: 62%; p < 0.05), 68% (83%; p < 0.01) of the patients were living in the catchment area at the time of the first contact. 82% (68%) of the patients were suffering from non-organic psychosis at the time of the first contact (p < 0.05). The average period between onset of disease and first therapeutical contact was 5 years (6 years; not significant). The average value of the PSR at first therapeutical contact is 2.6 (2.3; p < 0.01). The difference of average age when the mental disorder was diagnosed is highly significant (group with LSH: 27 years, total group: 30 years; p < 0.01), despite an elevated share of patients who fell ill for the first time between 45 and 59 years. LSH-patients thus suffered from an earlier or very late onset of the psychiatric disorder. When they fell ill, they did not come earlier or later for first treatment. Highly significant differences could be shown regarding the employment situation and primary income at the first therapeutical contact, with a disadvantageous situation for the LSH-group (p < 0.01). However, we found no significant differences regarding the living situation (p < 0.05).

## Discussion

There is always something arbitrary and abstract in trying to understand the individual stories of people's lives with scoring systems, especially regarding mentally ill people. But beside guidelines for clinical diagnosis and classification, we need suitable standards for community psychiatric care systems in order to prevent mentally ill patients from undergoing LSH.

Those patients who fulfilled our criteria for LSH were mostly suffering from psychotic disorders, were predominantly male and the onset of the disease usually occurred earlier than in the case of other chronically mentally ill clients. However, the first therapeutical contact after onset was not earlier or later than in other cases and also their living situation did not differ from the total group of evaluated patients [19]. Comparing the situation of employment [20] and primary income, however, the differences were significant and they imply that the group of LSH-patients were living under worse conditions than other patients even at the time of the first contact.

Our results corroborate a connection between an increased psychosocial risk and a disadvantageous course like LSH. A highly significant connection exists between the scoring system of PSR at the time of the first therapeutical contact and future LSH; i.e. a high psychosocial risk at the outset of treatment is linked to a high risk of LSH. The statistical weighting of these single features proved the bad influence of social disintegration, in particular unemployment. It seems that the characteristics of social disintegration are even more important than the characteristics referring to the disease. These results are consistent with those of other studies [7], according to which a good premorbid adjustment at work and good social relationships usually predict a positive course of the disease.

## Conclusion

The introduced PSR is seemingly a suitable instrument for registering features that lead to a higher risk of LSH. Special efforts are necessary to concentrate therapeutical awareness on this group of patients as early as possible. This study can help in developing community psychiatric care structures in those regions where outpatient efforts have not yet been established. The determination of a simple sum score makes it possible to calculate the risk of LSH on the basis of a small number of characteristics drawn from commonly registered psychiatric base data. This enables psychiatric services to concentrate on high-risk patients in order to avoid LSH.

## Competing interests

The author(s) declare that they have no competing interests.

## Authors' contributions

SB and HE conceived and designed the evaluation and helped to draft the manuscript. HE re-evaluated the clinical data and revised the manuscript. SB evaluated and performed the statistical analysis and revised the manuscript. SB, HE and PB collected the clinical data, interpreted them and revised the manuscript. MZ and WM re-analyzed the clinical and statistical data and revised the manuscript. All authors read and approved the final manuscript.
